# Reduced Need for Rescue Antiemetics and Improved Capacity to Eat in Patients Receiving Acupuncture Compared to Patients Receiving Sham Acupuncture or Standard Care during Radiotherapy

**DOI:** 10.1155/2017/5806351

**Published:** 2017-02-08

**Authors:** Anna Enblom, Gunnar Steineck, Mats Hammar, Sussanne Börjeson

**Affiliations:** ^1^Division of Physiotherapy, Department of Medical and Health Sciences, Linköping University, Linköping, Sweden; ^2^Department of Clinical Neuroscience, Osher Center for Integrative Medicine, Karolinska Institutet, Stockholm, Sweden; ^3^Department of Oncology-Pathology, Karolinska Institutet, Stockholm, Sweden; ^4^Division of Clinical Cancer Epidemiology, Department of Oncology, Sahlgrenska Academy, Gothenburg University, Gothenburg, Sweden; ^5^Division of Obstetrics and Gynecology, Department of Clinical and Experimental Medicine, Linköping University, Linköping, Sweden; ^6^Division of Nursing, Department of Oncology and Department of Medical and Health Sciences, Linköping University, Linköping, Sweden

## Abstract

*Objective. *To evaluate if consumption of emesis-related care and eating capacity differed between patients receiving verum acupuncture, sham acupuncture, or standard care only during radiotherapy.* Methods. *Patients were randomized to verum (*n* = 100) or sham (*n* = 100) acupuncture (telescopic blunt sham needle) (median 12 sessions) and registered daily their consumption of antiemetics and eating capacity. A standard care group (*n* = 62) received standard care only and delivered these data once*. Results. *More patients in the verum (*n* = 73 of 89 patients still undergoing radiotherapy; 82%, Relative Risk (RR) 1.23, 95% Confidence Interval (CI) 1.01–1.50) and the sham acupuncture group (*n* = 79 of 95; 83%, RR 1.24, CI 1.03–1.52) did not need any antiemetic medications, as compared to the standard care group (*n* = 42 out of 63; 67%) after receiving 27 Gray dose of radiotherapy. More patients in the verum (*n* = 50 of 89; 56%, RR 1.78, CI 1.31–2.42) and the sham acupuncture group (*n* = 58 of 94 answering patients; 62%, RR 1.83, CI 1.20–2.80) were capable of eating as usual, compared to the standard care group (*n* = 20 of 63; 39%).* Conclusion. *Patients receiving acupuncture had lower consumption of antiemetics and better eating capacity than patients receiving standard antiemetic care, plausible by nonspecific effects of the extra care during acupuncture.

## 1. Introduction

Many patients are interested in receiving acupuncture for a variety of symptoms during various types of cancer therapy [1], so also during radiotherapy [2], for example, emesis (nausea and vomiting) [3]. Acupuncture was seen to reduce emesis during radiotherapy [4] and chemotherapy [5] compared to standard care and in some studies also compared to sham acupuncture [5], but it is not known if acupuncture for emesis decreases consumption of emesis-related care and reduces negative consequences of emesis, for example, reduced eating capacity. Approximately 60–70 percent of patients were seen to experience nausea during abdominal or pelvic irradiation [3, 6]. If inadequately controlled, severe emesis may have clinical consequences, such as dehydration, electrolyte imbalance, and malnutrition. Care related to emesis thus includes, for example, intravenous nutrition in patients with reduced eating capacity, rescue antiemetic medications, need for sick transportations or hospitalising [7]. During chemotherapy, nausea was seen to have a negative impact on nutritional status [8]. In our previous study, we found that eating capacity was reduced in patients experiencing nausea during radiotherapy, compared to patients free from nausea [9]. It is well-known that the consumption of antiemetics and emesis-related care during chemotherapy is high and costly [10, 11]. In a study including 11,495 American outpatients receiving chemotherapy, the average emesis-related cost was 1855 US Dollars per patient and day with emesis [11]. According to our literature searches, there are no previous studies regarding consumption of emesis-related care during radiotherapy.

During fractioned radiotherapy, nausea may continue for several weeks and the long-term use of antiemetics may induce side-effects such as headache and constipation [6]. Antiemetics do not have satisfactory effects in all patients or are underused; in our previous study, one-third of patients with radiotherapy-induced nausea considered their antiemetic pharmacotherapy insufficient and three-quarters of the patients were interested in receiving acupuncture or in receiving more information regarding acupuncture for emesis [3]. There are indications that acupuncture in general compared to standard care may be a cost-effective therapy in a variety of other conditions [12], since the consumption of extra care decreased. Patients treated with verum acupuncture did not experience less emesis compared to patients treated with sham acupuncture [13], but acupuncture highly reduced emesis compared to standard care [4]. Even before the patient defines discomfort in the stomach area as nausea, the appetite and thus capacity to eat may decrease; it is not known if acupuncture affect patients' eating capacity. In contrast to the large number of studies describing consequences of chemotherapy-induced emesis [8, 10, 11], emesis-related care-consumption and eating capacity during radiotherapy is to our knowledge not previously described. The purpose was thus to evaluate if consumption of emesis-related care and eating capacity differed between patients receiving verum acupuncture, sham acupuncture, or standard care only for emesis during radiotherapy.

## 2. Material and Methods

### 2.1. Design

The study was approved by the regional ethics committee (02-420, M167-04) and by an external monitoring committee and had two parts. The first part* cross-sectionally* after a mean dose of 27 Gray (Gy) compared patients randomized to verum or sham acupuncture (the “needling groups”) and patients receiving standard care (the “standard care group”) regarding emesis-related care, that is, consumption of antiemetics and eating capacity. The second part* longitudinally* compared patients randomized to verum and sham acupuncture regarding consumption of emesis-related care, that is, consumption of antiemetics, hospitalisation, stays at patient hotels or sick transportations, and eating capacity.

### 2.2. Patients and Setting

**Figure 1:**
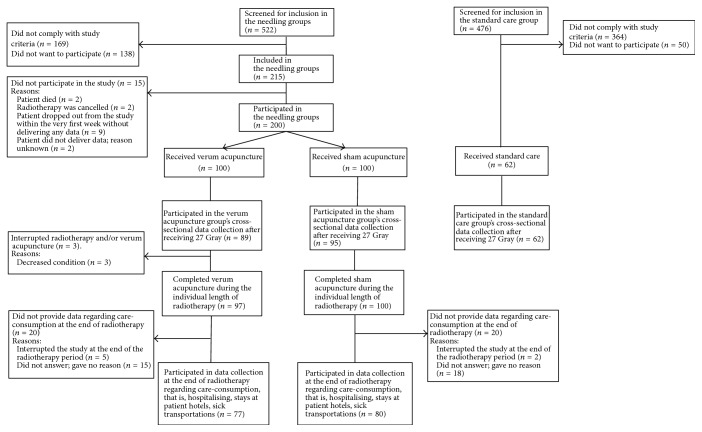
Number of patients included in the verum and sham acupuncture treatments and the data collection. The patients provided summed numbers of 10219 answers regarding antiemetic consumption and 10218 answers regarding eating capacity.

For the needling groups, we consecutively included patients at two Swedish University Hospitals during a three-year period (Figure 1). Inclusion criteria were an age of at least 18 years, a gynecologic, anal, rectal, colon, stomach, pancreatic, or testicular cancer, and a planned radiation over pelvic or abdominal fields of at least 800 cm^3^ volume and 25 Gy dose. Only patients with ability to give informed consent, and capability to take part in the treatment and data collection procedure were included, meaning that patients with very poor physical or mental condition were not included, for example, patients severely sedated and confused due to their cancer illness or mental illness. Exclusion criteria were antiemetic treatment or persistent emesis within 24 hours before start of radiotherapy (e.g., patients with constant emesis related to a vestibular neuritis were excluded, but not patients experiencing intermittent motion illness) or acupuncture therapy during the past year irrespective of indication or acupuncture therapy any time before for emesis. For the standard care group, we cross-sectionally during four different days included all patients receiving radiotherapy at one of the above mentioned University Hospitals, and also in another University Hospital, using the inclusion criteria mentioned above.

We screened 522 and 476 patients for participation in the needling groups and the standard care group, 169 and 363 did not meet study criteria, 138 and 50 did not want to participate, and 215 and 63 patients were originally included. Of these 215 patients, 15 did not participate, while 200 patients participated in the needling groups. At the time for the cross-sectional comparison after receiving mean 27 Gy, 184 out of the 200 patients were still undergoing radiotherapy (89 in the verum and 95 in the sham acupuncture group) (Figure 1), while 16 patients had finished radiotherapy due to the individual length of the therapy. One out of the 95 patients in the sham acupuncture group did not deliver data regarding eating capacity while all 89 and 63 patients in the verum acupuncture group and standard care group delivered data.

### 2.3. Procedure

Patients received fractioned radiotherapy, one fraction per day (Mondays to Fridays) for a median of five weeks. The patients were mostly treated as outpatients. If needed because of their cancer, medical condition, or severe side-effects, for example, emesis, they were treated as inpatients according to the ordinary clinical routines. The outpatients travelled to the radiotherapy department (a distance between 0 and 290 kilometres) by bus or car. If needed due to their medical condition, they used sick transportation. If the outpatients could not travel daily, they stayed at patient hotels.

The patients in the needling groups were informed orally and in written form that the beneficial effect of either treatment was not known and that “You will receive—without being told which—an ordinary acupuncture treatment with needles penetrating the skin or another treatment with needles placed just against the skin.” None of the randomization alternatives were cited as a “sham” or “placebo” treatment. A coordinating nurse, not involved in the needling, randomized the blinded patients stratified for gender and hospital to verum or sham acupuncture by using a computer-based randomization table. The evaluator (first author) and all healthcare professionals other than the acupuncture-providing therapists were blind to the treatment allocation. The standard care group knew, of course, that no acupuncture was given. They had been informed that the aim of the data collection was to investigate emesis during radiotherapy and its potential consequences.

### 2.4. The Healthcare Professionals and Care Routines

Oncologists not involved in the study prescribed rescue antiemetics if needed in both the needling groups and the standard care group, as suggested in international guidelines [14]. They prescribed antiemetics according to standardised ordinary clinical routines, at doses based on the Swedish Medicine Engine Information (http://www.fass.se) (Table 1). Oncology nurses identified potential need for extra care related to emesis, for example, the need for intravenous nutrition, according to ordinary clinical routines. In the needling groups, seven physiotherapists performed verum or sham acupuncture according to a standardised protocol. They started 30 minutes verum or sham acupuncture treatments the first day of radiotherapy and continued three times/week for the first two weeks, followed by twice/week for the remaining radiotherapy period (Mondays through Fridays), in line with suggested clinical routines to obtain long-term effects [15]. The physiotherapists were educated and experienced in the methods for practicing verum and sham acupuncture. They maintained everyday conversations with the patients but avoided the subject emesis. One to three patients were treated simultaneously in different rooms. For detailed information regarding the physiotherapists' education and previous experiences of practicing acupuncture, please see our previous report [13].

### 2.5. The Needling Groups

The physiotherapists delivered western medical manual verum acupuncture bilaterally to the standard antiemetic point pericardium six (PC6) [16] between the tendons of palmaris longus and flexor carpi radialis at two body-inches (one body-inch = one cun: the acupuncture-specific dimension equivalent to the greatest width of the individual patients' thumb,* approximately* 1.5 cm) proximal to the wrist. Sharp needles in stainless steel (manufactured by DongBang, Korea), diameter 0.25 × length 40 millimeters, were inserted to a depth of a half body-inch. The physiotherapists manipulated the needles three times (at the start, middle, and end of every session) by twirling and lifting until “deqi” occurred. “Deqi” was reached when the patient reported a sense of numbness or soreness and the physiotherapist noted a minimal muscular contraction around the needle [13].

The physiotherapists delivered sham acupuncture bilaterally to a nonacupuncture point two body-inches proximal to PC6 with the nonpenetrating telescopic sham needle “Park's sham device” [17], 0.25 × 40 millimeters (fully extended length). The credible [18, 19] sham acupuncture needle (stainless steel, manufactured by DongBang, Korea) looks identical to a real needle but is blunted and glides upwards into its handle instead of penetrating, giving an illusion of penetration. “Park's sham devise” marking tube with a bottom plate covered with double-sticky tape marked the needling-points in both groups, and holds the sham needle in place. The therapist manipulated the sham needles a few seconds three times per session resulting in that the needles touched the skin, but no “deqi” occurred [20]. Except for placing and manipulating the needle, the sham needle was not pressed against the skin at all (i.e., no “acupressure”) [16].

### 2.6. Data Collection

We in both the needling groups and the standard care group collected data on gender, age, tumor diagnosis, and dose of radiotherapy in the medical records, and the patients provided sociodemographic data and the data described below, using study-specific questions. The questions were developed from patient interviews, were tested for validity and reliability and were used in a pilot study [21]. This methodology of developing and validating study-specific questions [22] has been used in over 80 publications [22–24].

#### 2.6.1. Measurement of Emesis-Related Care

The patients in the needling groups every morning during the entire radiotherapy period (Mondays to Sundays) in writing answered the questions: “Have you within the previous 24 hours experienced nausea?” (“Yes” or “No”) and “Have you within the previous 24 hours been taking any medications for emesis (on prescription or without prescription)?” (“Yes” or “No”). The patients detailed the names and doses of the medications taken. The patients in the standard care group answered the same questions at the cross-sectional data collection.

The day after the last radiotherapy fraction had been given, only the patients in the needling groups answered also the written questions: “Have you any time within the radiotherapy period* due* to nausea or vomiting…: “…been hospitalised?”, “…choose to stay in the patient hotel instead of your usual living?”, and “…needed sick transportation (with taxi) to or from the radiotherapy department?” The patients answered the questions by “No” or “Yes, for the specific dates…” (the actual dates were specified).

#### 2.6.2. Measurement of Eating Capacity

The patients in the needling groups every seventh morning during the entire radiotherapy period answered the question: “Have you been capable of eating as much as you are used to?” using the answering alternatives: “Yes,” “Yes, I have been eating more,” “No, I have been eating less,” “No, I have been eating much less,” or “No, I have been eating much less; I needed intravenous nutrition.” If the patients choose the last alternative, they also detailed the number of days with intravenous nutrition. The patients in the standard care group answered the same question at the cross-sectional data collection, using the two answering alternatives “Yes” or “No, I have been eating less.”

### 2.7. Statistical Analyses

We compared the verum and sham acupuncture group and the standard care group regarding the clinical and sociodemographic variables seen in Table 2. Chi-square test (nominal variables with *n* more than five per category) or Fisher's exact test (diagnosis), Mann-Whitney *U* test regarding ordinal variables (*n* of previous nausea situations), and Student's *t*-test regarding continuous variables (age) were used.

After the patients had received a mean (*m*) radiotherapy dose of 27 Gy, we cross-sectionally compared the number (*n*) and proportion (%) of patients who experienced nausea and who used any type of antiemetic medication within the past 24 hours in the needling groups and the standard care group using Fisher's exact test, presented as Relative Risks (RR) with 95 percent Confidence Intervals (CI). We cross-sectionally compared eating capacity between the groups (two-group comparison) using Mann-Whitney *U* test.

In the needling groups only, we calculated *n* and percent who used different types of antiemetic medications at least once within the longitudinal radiotherapy period and compared the verum and sham group using Fisher's exact test, presented with RR and CI. If a difference occurred, we then compared the needling groups regarding number of days consuming the antiemetic medication (continuous but not normally distributed variables) with Mann-Whitney *U* test. We calculated *n* of patients and *n* of days with need for hospitalisation, stays at patient hotels, or sick transportations due to emesis.

Eating capacity in the verum and sham acupuncture group was compared using Mann-Whitney *U* test. Median number of days that the patients in the needling groups had been eating as usual, less, and much less during the entire radiotherapy period was calculated, and Chi-square test compared the verum and the sham acupuncture group regarding proportion of patients who at least once answered that he/she had been eating much less and needed intravenous nutrition. In the needling groups (verum and sham acupuncture group), the Wilcoxon sign test (paired samples, ordinal variable) compared the capacity to eat between the start and the end of radiotherapy, and between the end of radiotherapy and four weeks after radiotherapy, respectively. The Statistical Package for the Social Sciences for Windows version 23.0 was used. The significance level was set at 5 percent; *p* values of ≥0.05 were considered as not statistically significant (ns).

## 3. Results

### 3.1. Patient Characteristics

Most patients in both the needling groups and the standard care group were women, at an age of more than 60 years, who received radiotherapy for a gynecological tumor (Table 2). There were no differences in sociodemographic or clinical variables between the verum acupuncture group, the sham acupuncture group, and the standard care group, except for the fact that the standard care group comprised more patients with a testicular tumor and thus more men than the needling groups. The patients in the needle groups were successfully blinded, as presented previously [13].

### 3.2. Consumption of Emesis-Related Care: Antiemetics

**Figure 2:**
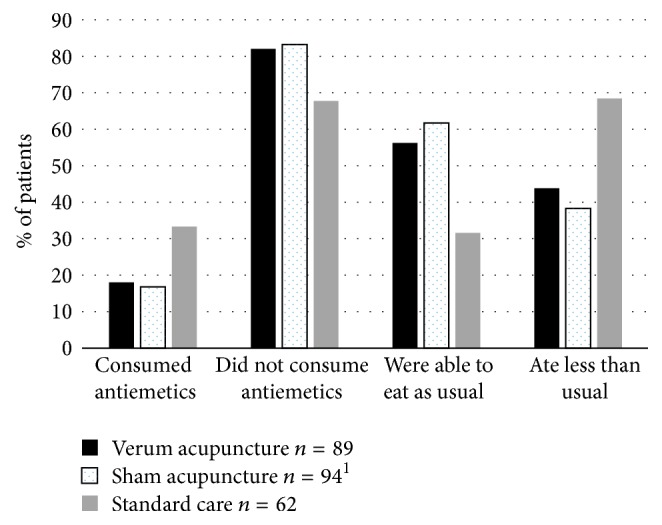
Proportion (%) of patients consuming antiemetics or not (left) and with capacity to eat as usual or not (right). Measured in patients in the verum acupuncture group; *n* = 89, sham acupuncture group; *n* = 95 (^1^*n* = 94 graded eating capacity), and standard care group; *n* = 62, when all three groups had received 27 Gray (mean) dose of radiotherapy.

At the cross-sectional comparison after receiving mean 27 Gy, more patients in both the verum and sham acupuncture group were free from nausea (67 of 89; 75% and 77 of 95; 81%) and did not consume any antiemetic medication (*n* = 73 out of 89; 82% (RR 1.23, CI 1.01–1.50) and 79 out of 95; 83% (RR 1.23, CI 1.01–1.50)), as compared to the standard care group (*n* = 42 out of 63; 68%). The verum and the sham acupuncture group did not statistically differ regarding consumption of antiemetics (RR 1.01, CI 0.88–1.16) (Figure 2).

According to the longitudinal analyses, 58 out of 100 (58%) patients in the verum acupuncture group and 63 out of 100 (63%) patients in the sham acupuncture group did not need to consume any antiemetic medication within the entire radiotherapy period (ns). More patients in the verum acupuncture group (87 of 100; 87%) than in the sham acupuncture group (75 of 100; 75%, RR 1.16, CI 1.01–1.33) did not consume corticosteroids. However, as presented in Table 3, number of days with consumption did not differ (*p* = 0.345).

### 3.3. Consumption of Emesis-Related Care: Hospitalising and Sick Transportations

Hospitalising related to emesis was needed in two patients in the verum acupuncture group (for 1 and 8 days, resp.) and in two patients in the sham acupuncture group (for 1 and 16 days, resp.). Four patients in the verum acupuncture group and three in the sham group stayed at the patient hotel instead of in their ordinary dwelling at any time within the radiotherapy period due to emesis (range 5–44 days and 1–32 days). Three patients in both the verum (for one day each) and in the sham (range 2–8 days) acupuncture group needed sick transportation due to nausea.

### 3.4. Eating Capacity

At the cross-sectional comparison, more patients in both the verum (*n* = 50 out of 89; 56%; RR 1.78, CI 1.31–2.42) and the sham acupuncture group (*n* = 58 out of 94; 62%; RR 1.83, CI 1.20–2.80) were capable of eating as usual, as compared to the standard care group (*n* = 20 out of 63; 39%). The verum and the sham acupuncture groups did not statistically differ (RR 1.12, CI 0.83–1.50) (Figure 2).

**Figure 3:**
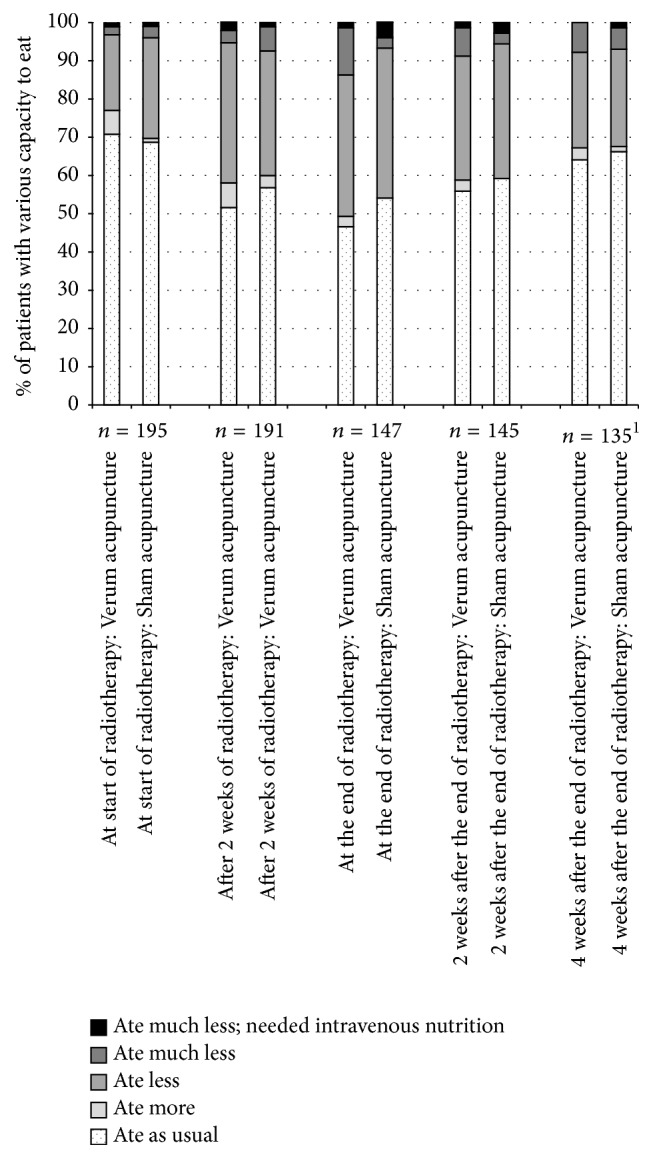
Proportion (%) of patients with different levels of eating capacity during and after radiotherapy in the verum and the sham acupuncture group. The number of patients decreases over time due to the individual length of radiotherapy. ^1^Two patients did not answer; reason unknown.

At the start of radiotherapy, 136 of 195 (70%) answering patients in the needling groups were capable of eating as usual. The capacity to eat decreased with time (*p* < 0.001); at the end of the radiotherapy period 74 of 147 answering patients (50%) were capable of eating as usual. Eating capacity improved with time after the end of radiotherapy (*p* < 0.001); four weeks after the end 88 of 135 answering patients (65%) were capable of eating as usual. As presented in Figure 3, there were no differences in gradings of eating capacity between the verum and sham acupuncture groups. In the verum group (*n* = 100) and the sham group (*n* = 100), 40 (40%) and 40 (40%) had at least at one time-point been eating much less, and 9 (9%) and 6 (6%) had been eating much less and did need intravenous nutrition (RR 1.50, CI 0.55–4.06). The number of days with various capacity to eat is presented in Table 4.

## 4. Discussion

This study found that patients receiving verum or sham acupuncture had lower consumption of antiemetics and better eating capacity than patients receiving standard antiemetic care only. The capacity to eat decreased during the radiotherapy period without differences between verum and sham acupuncture treated patients. Emesis-related care, other than antiemetic medications, was rarely needed.

Our findings may be interpreted as if the nonspecific components, including placebo-effects, of the needling procedure were effective to reduce the need for antiemetics in patients treated with radiotherapy while the specific characteristics of verum acupuncture were not effective. Specific components of genuine acupuncture may be penetration and stimulation of the skin at traditional acupuncture points [16]. Most medical treatments include components that may be divided into a specific treatment component, for example, a pharmacological substance, and a nonspecific component that includes the context surrounding the delivery of treatment [25]. The nonspecific treatment component is typically conveyed via the patient-clinician interaction and the context for treatment [26]. Our verum and sham acupuncture treated patients received extra care in terms of contact with a physiotherapist during needling, although the physiotherapist often treated two to three patients simultaneously, and the patients rested and often relaxed during the needling sessions. A wide range of studies of placebo treatments have elucidated pathways through which placebo-effects can be activated, often described as an interaction between psychological processes (e.g., expectations, hope) and neurobiological mechanisms (e.g., endocrine and immune functions) [27, 28]. Both the verum and the sham acupuncture group strongly believed that they received a genuine effective treatment, while the standard care group received no extra treatment and thus probably had lower expectations to stay unaffected from nausea and to be capable of eating as usual. High expectations, in terms of belief in the efficacy of acupuncture on the treated outcome, highly modified the effect in previous acupuncture interventions in a variety of settings [29]. That the verum and sham acupuncture groups were compared with a cross-sectional standard care group in our study, not to a third randomized arm, required for a thorough investigation of potential imbalance of confounding factors between the groups. Only testicular tumor patients and thus men were more frequent in the standard care group than in the needling groups. However, actually male gender reduces the risk for radiotherapy-induced emesis [3, 6]. The design may have the benefit of avoiding the impact of the patient information or the data collection per se on reported outcomes. Expectations to receive or not to receive an effective treatment [26, 29], as well as repeated measurement of outcomes per se, may reduce (through the so called Hawthorne effect) or increase emesis experience.

In this study, we evaluated if manual western acupuncture of the frequently used antiemetic point PC6 [16] reduced consumption of emesis-related care. Every condition treated with a certain type of acupuncture needs its own evaluation, and based on findings from our study we are unable to predict the effects of other kinds of acupuncture such as electroacupuncture or using more seldom used [16] antiemetic acupuncture points. In previous studies, manual acupuncture did not reduce nausea more than sham using a telescopic nonpenetrating needle, during neither chemotherapy (*n* = 80) [30] nor radiotherapy (*n* = 215) [4]. For chemotherapy-induced nausea, electroacupuncture of the point K1 on the heel did not reduce nausea or vomiting more than electroacupuncture on a sham-point (*n* = 103) [31]. However, K1 is not a traditional antiemetic point [16]. Electroacupuncture of PC6 reduced vomiting more than either simulated electroacupuncture with superficially inserted needles or standard care (*n* = 104) [5]. However, data indicate that the blinding of the patients may have failed. During chemotherapy, manual (*n* = 361) [32] or electroacupressure (*n* = 96) [33] and manual acupressure combined with manual acupuncture (*n* = 27) [34] both failed to reduce nausea more than a simulation of electroacupressure. However, manual acupressure (*n* = 160) reduced nausea and vomiting more than both sham-points and standard care [35]. Chemotherapy patients (*n* = 70) [36] and radiotherapy patients (*n* = 277) [4] receiving acupuncture experienced less nausea compared to patients receiving standard care, including antiemetics alone. Chemotherapy patients (*n* = 233) [37] and (*n* = 90) [38], and radiotherapy patients (*n* = 88) [39] receiving acupressure, and chemotherapy patients (*n* = 94) receiving combined acupuncture and acupressure [40], all had larger reductions of nausea compared to patients receiving standard care. Accordingly, the results of different studies vary a lot and indicate large nonspecific effects. In a recent study regarding relaxing effects of acupuncture (*n* = 243), individuals who received positive communication regarding expected treatment effects doubled their treatment expectations as compared to individuals receiving neutral communication and the individuals with high expectation doubled the improvement in relaxation as compared to individuals with low expectations [41]. Irrespective of the fact that if the benefit for the patients was based on nonspecific or specific effects of acupuncture, the needling seemed beneficial for the patients. This is in line with results regarding acupuncture in general; a review of acupuncture for various pain conditions, indicate benefit for patients but larger nonspecific than specific effects of acupuncture [42].

The fact that more patients in both the verum and the sham acupuncture group were capable of eating as usual, as compared to the standard care group, is a new observation. According to our literature search (https://www.pubmed.gov, search terms radiotherapy and nutrition, or radiotherapy and eating, date 1st September 2016), there are no previous studies describing eating capacity in cancer patients irradiated over abdominal or pelvic fields. The better eating capacity seen in the needling groups was probably valuable for the patients; only 66 percent of nauseous patients were able to eat as usual during radiotherapy as compared to 84 percent of patients free from nausea in a previous study [9]. Regarding the more studied chemotherapy-induced nausea, Farrell and coworkers [8] in 104 patients observed that severe nausea had a negative impact on nutritional status. Improved eating capacity and thus nutrition status seems very beneficial for patients undergoing emetogenic cancer therapy [7].

The need for hospitalising, stays in patient hotels, and sick transportations related to emesis was low in patients treated with both verum and sham acupuncture, probably due to the fact that radiotherapy-induced nausea often is of low intensity. In previous studies, the intensity of radiotherapy-induced nausea was graded as mild in 60 to 72 percent of nauseous patients [3, 4, 13]. Although the long-term nausea during radiotherapy is bothersome for the patients in terms of reduced quality of life [3, 9], mild nausea possibly less often causes need of extra care, in contrast to chemotherapy-induced nausea [10, 11]. That the consumption of emesis-related care did not differ between the verum and sham acupuncture group may be interpreted as that verum acupuncture does not affect consumption of emesis-related care during radiotherapy, or that a “floor-effect” was seen. We do not know if the need for hospitalising, stays in patient hotels, and sick transportations related to emesis would have been higher if no needling had been given, since we did not collect these data in the cross-sectional standard care group.

Strengths of the study are not only the high compliance with data collection and the thorough control of reasons for cancellations of therapy and of potential imbalance between the needling groups and the standard care group, but also the large number of studied patients as compared to many other studies on emesis [16]. Without the thorough control of potential imbalance between the needling groups and the standard care cohort, a weakness of our study would have been the cross-sectionally performed comparisons. When interpreting the results of an acupuncture study, it is important to take the reasons for cancelling into consideration. Hypothetically, some patients in the sham acupuncture group could have cancelled because of* occurrence of *emesis leading to hospitalising, whereas patients in the verum acupuncture group could have cancelled because of* lack of* emesis (the patients may find it unnecessary with further needling) leading to the lack of difference between the groups. However, this was not true in our study, since cancelling were related to other factors than emesis. At the data collection at the end of radiotherapy, 15 patients in the verum and 18 patients in the sham acupuncture group did not deliver data. It accordingly seemed more difficult for the patients to comply with data collection when they did no longer had contact with the needling therapist and the study coordinator. Other strengths of the study are the sham-controlled design using the nonpenetrating sham needle which is as both credible and inert as possible [19]. Of course the telescopic sham needle touched the skin during the few seconds for placing and manipulating the sham needles every needling session. However, the sensorimotor activation of a variety of brain regions was clearly lower during sham needling as compared to during verum acupuncture needling [20]. We choose to measure care-consumption and eating capacity using pretested [21] and previously used [9] single item questions, adopting a methodology [22] developing questions those patients find meaningful and not too bothering [24]. The choice of using single questions was based on a wish to reduce the patient burden compared to a longer psychometric questionnaire, which seemed important since some of the questions were repeated daily. The eating capacity question seemed to be enough sensitive, since it in a previous study discriminated between patients with and without nausea [21]. We made no power-calculation for the outcomes emesis-related care-consumption and eating capacity. However, the study met the preformed criteria for statistical power regarding the primary endpoint that our previous main study [13] was primary designed for; occurrence of nausea (*n* ≥ 200 patients, effect size 20%, 80% power at 5% significance level).

Since nonspecific antiemetic treatment effects during the needling caring situation seem to have achieved the fact that both patients receiving verum and sham acupuncture needed less antiemetics and had larger capacity to eat compared to patients receiving standard care, further studies should evaluate the effect of contextual caring factors for strengthening the quality of care and reducing side-effects such as emesis during cancer therapy.

## Figures and Tables

**Table 1:** Routines for antiemetic prescriptions.

Variable	Antiemetic type	Name and dose
At *first* occurrence of mild nausea, prescribe^1^	Antihistamine with antiemetic effect	Meclozine 25 mg 1-2/day
At *first* occurrence of severe nausea or occurrence of vomiting, instead prescribe^1^ *or* if not satisfactory nausea control, *also* add^1^	Serotonin-receptor antagonists	Ondansetron 8 mg 2/day *or* granisetron 1 mg 1/day *or* tropisetron 5 mg 1/day
If not satisfactory nausea control, *also* add^1^	Corticosteroids	Betamethasone 4 mg 2/day

Description of the standardised clinical routines for prescription of antiemetic medications. mg = milligram. ^1^If the patient agreed to consume medications.

**Table 2:** Clinical characteristics of the patients in the verum acupuncture, sham acupuncture, or standard care group.

Characteristics	Needling groups *n* = 200		Standard care group *n* = 62
	Verum acupuncture *n* = 100	Sham acupuncture *n* = 100	
*Tumor diagnosis n* (%)			
Gynecological	66 (66)	71 (71)	37 (60)
Colon/rectal	28 (28)	27 (27)	11 (18)
Testicular	2 (2)	0 (0)	6 (10)
Pancreas, stomach, or gallbladder	4 (4)	2 (2)	8 (13)
*Total radiotherapy dose* (Gray) mean ± SD	47.9 ± 10.7	50.3 ± 10.3	41.8 ± 10.0
*Concomitant chemotherapy*, *n* (%)		*n* = 99	*n* = 61
Yes	28 (28)	29 (29)	15 (25)
No	72 (72)	70 (71)	46 (75)
*Medication for any other illness/symptom* ^1^, *n* (%)	*n* = 99	*n* = 100	*n* = 62
Yes	80 (80)	88 (88)	40 (65)
No	19 (19)	12 (12)	22 (35)
*Gender n* (%)			
Man	18 (18)	14 (14)	19 (31)
Woman	82 (82)	86 (86)	43 (69)
*Age* years: mean ± SD	64 ± 13.8	63 ± 13.9	63 ± 14.5
19–40	7 (6)	6 (6)	6 (10)
41–60	34 (31)	34 (32)	17 (27)
61–89	68 (62)	66 (62)	39 (63)
*Labor status n* (%)			*n* = 62
Employed	33 (33)	38 (38)	21 (34)
Retired/sickness pension	65 (65)	57 (57)	26 (42)
Other	2 (2)	4 (4)	15 (24)
*Education level n* (%)	*n* = 94	*n* = 98	
Elementary school	41 (44)	53 (54)	27 (44)
Secondary school	29 (31)	26 (27)	17 (27)
University	24 (26)	19 (19)	18 (29)
*Previous nausea n* (%)			
*During previous chemotherapy*	*n* = 96	*n* = 98	
Not relevant	55 (57)	58 (60)	43 (69)
No	11 (11)	12 (12)	15 (24)
Yes	30 (31)	28 (29)	4 (6)
*During pregnancy*	*n* = 89	*n* = 92	*n* = 61
Not relevant	26 (29)	28 (30)	33 (54)
No	19 (21)	24 (26)	6 (10)
Yes	44 (49)	40 (43)	22 (36)
*In any previous situations* ^2^	*n* = 96	*n* = 98	*n* = 61
No	22 (23)	29 (30)	17 (27)
Yes	74 (77)	69 (70)	44 (72)
*N of previous nausea situations* ^2^, md (25th–75th percentile)	*n* = 97 2 (1–3)	*n* = 98 2 (1–3)	*n* = 61 2 (0–3)
0–2 situations	68 (70)	67 (68)	44 (71)
3–5 situations	29 (30)	31 (32)	18 (29)

Numbers (*n*) of patients answering the questions are presented. ^1^For other conditions than emesis. ^2^In travelling, unpleasant smells/sights, anxiety, chemotherapy, or pregnancy. SD = standard deviation. Md = median.

**Table 3:** Consumption of medications for emesis in patients treated with verum acupuncture, sham acupuncture, or standard care during radiotherapy.

Variable	Verum acupuncture group	Sham acupuncture group	Standard care group
*Consumption of antiemetic types at the cross-sectional comparison n* (%) of patients consuming a variety of types of antiemetics^2^	*n* = 89	*n* = 95	*n* = 62
Serotonin-receptor antagonists	4 (4)	5 (5)	1 (2)
Dopamine-receptor antagonists	7 (8)	5 (5)	18 (29)
Corticosteroids	3 (3)	4 (4)	0 (0)
Other^1^	8 (9)	9 (9)	2 (3)
No antiemetics	67 (75)	72 (81)	42 (68)
*Consumption of antiemetic types within the entire radiotherapy period n* (%) of patients consuming a variety of types of antiemetics at least once^2^, mean number days of consumption ± standard deviation	*n* = 100	*n* = 100	*n* = 62
Serotonin-receptor antagonists	21 (21) 8.7 ± 8.4	23 (23) 7.8 ± 6.9	—
Dopamine-receptor antagonists	24 (24) 8.8 ± 9.7	21 (21) 7.5 ± 7.4	—
Corticosteroids	13 (13) 12.0 ± 6.0	25 (25) 10.6 ± 8.2	—
Other^1^	12 (12) 11.6 ± 11.6	9 (9) 8.0 ± 10.6	—
No antiemetics	58 (58)	63 (63)	—

Numbers (*n*) of patients answering the questions are presented. *n* was 89 of the 100 verum acupuncture treated patients and 95 of the 100 sham acupuncture treated patients at the cross-sectional comparison (after receiving mean 27 Gray), since 11 of the acupuncture treated and 5 of the sham treated patients had finished radiotherapy due to the individual length of therapy. Md = median. ^1^Other types were antihistamines, omeprazole, or medications for anxiety, used against emesis. ^2^The patients could consume more than one type of antiemetics. — = consumption was not registered longitudinally in the standard care group.

**Table 4:** Eating capacity in patients treated with verum or sham acupuncture during radiotherapy.

Variable	Total	Verum acupuncture group	Sham acupuncture group
*Eating capacity within the entire radiotherapy period *md, 25th–75th percentile of summed number of days	*n* = 200	*n* = 100	*n* = 100
*Ate as usual*	16, 4–30	15, 2–30	16, 11–30
*Ate less*	10, 2–21	12, 0–22	10, 4–20
*Ate much less*	1, 0–7	1, 0–6	1, 0–7

The summed number of days with different capacity to eat during the entire radiotherapy period is presented.
